# Aflibercept 5+PRN with retinal laser photocoagulation is more effective than retinal laser photocoagulation alone and aflibercept 3+PRN with retinal laser photocoagulation in patients with high-risk proliferative diabetic retinopathy and diabetic macular edema: a 12-month clinical trial

**DOI:** 10.3389/fendo.2024.1286736

**Published:** 2024-02-22

**Authors:** Shuting Li, Yuan Tao, Mengyao Yang, Hui Zhao, Mingwei Si, Wenxuan Cui, Hong Wang

**Affiliations:** ^1^ Department of Ophthalmology, Qilu Hospital of Shandong Province, Jinan, China; ^2^ Department of Ophthalmology, The Second People’s Hospital of Jinan, Jinan, China

**Keywords:** aflibercept, retinal laser photocoagulation, diabetic retinopathy, cytokines, microaneurysm

## Abstract

**Objective:**

This study aimed to investigate and compare the efficacy and safety of retinal laser photocoagulation (PRP) alone, PRP with aflibercept 3+PRN, and PRP with aflibercept 5+PRN in patients with both high-risk proliferative diabetic retinopathy (PDR) and diabetic macular edema (DME).

**Methods:**

Overall, 170 patients with high-risk PDR and DME (170 eyes from 170 patients) who visited our ophthalmology clinic from December 2018 to December 2020 were divided into the PRP (n=58), aflibercept 5+PRN with PRP (n=53), and aflibercept 3+PRN with PRP (n= 59) groups. General information, such as age, sex, and eye category, was obtained. Moreover, best-corrected visual acuity (BCVA), baseline central macular foveal thickness (CFT), microaneurysm (MA), area of neovascularization (NV), area of hard exudate (HE), and cytokine levels in atrial fluid before and after treatment, were assessed. The χ^2^ test was used for comparison between groups for statistical data. Analysis of variance was used for the statistical description of measurement data, independent samples were analyzed using Student’s *t*-test, and Student–Newman–Keuls test was used for group comparisons. Differences were considered statistically significant at P < 0.05.

**Results:**

After treatment, no significant improvement in the BCVA (logMAR) of patients in the PRP group was observed. The BCVA (log MAR) decreased from 0.72 ± 0.17 and 0.74 ± 0.17 to 0.50 ± 0.13 and 0.53 ± 0.17 in PRP with aflibercept 5+PRN and PRP with aflibercept 3+PRN groups, respectively, with a statistically significant difference compared to those in the PRP group (P<0.05 in all cases). However, no statistically significant difference was observed between the combined treatment groups (P>0.05). The CFT in the PRP-only group decreased slightly from 361.80 ± 36.70 μm to 353.86 ± 40.88 μm, with no statistically significant difference (P>0.05), whereas the CFT in the aflibercept 5+PRN with PRP and aflibercept 3+PRN with PRP groups decreased from 356.57 ± 37.57 μm and 358.17 ± 44.66 μm to 284.87 ± 31.52 μm and 303.19 ± 37.00 μm, respectively, with statistically significant differences before and after treatment (P<0.05 for both groups). Statistically significant differences were observed in CFT between the three groups after treatment (P<0.05 in all cases). The number of MA (pcs) in the PRP, aflibercept 5+PRN with PRP, and aflibercept 3+PRN with PRP groups decreased from 118.34 ± 27.96, 118.60 ± 33.34, and 116.59 ± 28.95 to 92.95 ± 29.04, 44.60 ± 20.73, and 54.26 ± 25.43, respectively. The two-way comparison of the three groups revealed statistically significant differences in MA (P<0.05 in all cases). In the three groups, NV decreased from 1.00 ± 0.21 mm², 1.01 ± 0.18 mm², and 0.98 ± 0.20 mm² before treatment to 0.49 ± 0.17 mm², 0.31 ± 0.16 mm², and 0.38 ± 0.14 mm², respectively, with statistically significant differences (P<0.05 in all cases). After 12 months of treatment, 13, 18, and 18 patients had reduced HE area in the PRP-only, aflibercept 5+PRN with PRP, and aflibercept 3+PRN with PRP groups, respectively, with statistically significant differences (P<0.05 in all cases). After 12 months of treatment, vascular endothelial growth factor, monocyte chemoattractant protein-1, and glial fibrilliary acidic protein levels (pg/mL) in the aqueous humor decreased in both combined treatment groups compared with that at baseline, with statistically significant differences; however, no significant difference was observed between the two combined treatment groups (P>0.05).

**Conclusion:**

Aflibercept 5+PRN combined with PRP was safe and effective in treating patients with high-risk PDR and DME, and was more effective than PRP-only and aflibercept 3+PRN with PRP in improving CFT and MA.

## 1 Introduction

Diabetes mellitus (DM) is a metabolic disease predominantly characterized by hyperglycemia. Generally, DM is caused by insufficient insulin secretion in the body; however, the other biological mechanisms remain unclear. Long-term illness in patients with DM damages various organs in the body, such as the eyes, kidneys, and heart, seriously affecting organ function. Nowadays, the quality of life of people has improved significantly, eating habits have changed, sugar intake is increasing, and the number of patients with DM is increasing. Statistics show that in 2017, the number of patients with DM worldwide reached 425 million (aged 20–79 years), which will exceed 600 million in 30 years; moreover, patients in low- and middle-income countries, such as China and India, account for 80 percent of the total DM population (1). According to the WHO, patients with DM worldwide increased to 366 million in 2011, which is expected to increase to 500 million in 2025, with more than 150 million patients experiencing ocular complications, such as diabetic retinopathy (DR) (2, 3). DR is a form of ocular microangiopathy and the most serious DM-related complication; it seriously endangers the health of patients with DM (4). DR pathogenesis includes increased endothelial cells in the eye capillaries, increased intimal thickness, damaged pericytes, microangioma, and damaged blood-retina barrier due to increased permeability of the blood vessels, microvascular obstruction, and neovascularization (NV) (5, 6). Currently, the prevalence of DR is 34.6% worldwide; however, it is higher in some developed countries, reaching 40.3% (7). The proportion of patients with type 1 and 2 DM suffering from blindness due to DR is 3.6% and 1.6%, respectively (8). DR is associated with significantly reduced living standards, huge medical costs, and increased social burden (9, 10).

Many anti-vascular endothelial growth factor (VEGF) drugs exist; however, the use of therapeutic drugs is strictly controlled. The main drugs recommended for treating DM-related visual complications are ranibizumab and aflibercept. However, the aflibercept 3 + PRN or 5 + PRN program is less effective for treating DR worldwide. Determining a standardized loading dose and administration interval for the intravitreal injection of aflibercept for DR treatment is challenging. In cases of deteriorated retinal anatomy and function, the administration interval needs to be shortened. Therefore, this study aimed to compare the safety and efficacy of retinal laser photocoagulation (PRP) and PRP with intravitreal injection of aflibercept (3 + PRN or 5 + PRN) for patients with high-risk proliferative DR (PDR).

## 2 Materials and methods

### 2.1 Study participants

#### 2.1.1 Population data statistics

This study included 170 ophthalmology outpatients diagnosed with high-risk PDR through detection techniques, such as visual acuity, intraocular pressure, slit lamp, fundus photography, ocular B ultrasound, optical coherence tomography (OCT), and fundus fluorescence angiography at the Qilu Hospital of Shandong University between December 2018 and December 2020. According to their treatment regimen, the patients were divided into the laser (n=58; men: women=30:28; mean age=66.7 ± 3.9 years; right eye=29 [50%]; left eye=29 [50%]), aflibercept 5 + PRN intravitreal injection with PRP (n=53; men: women=28:25; mean age=65.2 ± 4.5 years), and aflibercept 3 + PRN intravitreal injection with PRP (n=59; men: women=28:31; age=67.8 ± 3.7 years; right eye=27 [50.9%]; left eye= 26 [49.1%]) groups. Each group was followed up for 12 months (**Table 1**). This study was conducted in accordance with the Declaration of Helsinki and approved by the Medical Ethical Review Committee of Qilu Hospital of Shandong University (Approval Letter No.: 2019091). All the patients provided written informed consent.

**Table 1:** General Patient Data.

	PRP group (n =58)	Aflibercept 5 + PRN with PRP group (n =53)	Aflibercept 3 + PRN with PRP group (n =59)	*t, χ^2^*	*P*
**Man**	30 (51.7%)	28 (52.8%)	28 (47.5%)	0.368	0.832
**Woman**	28 (48.3%)	25 (47.2%)	31 (52.5%)		
**Age (year)**	66.7 ± 3.9	65.2 ± 4.5	67.8 ± 3.7	2.550	0.081
**O. D**	29 (50.0%)	27 (50.9%)	29 (49.2%)	0.036	0.982
**L. E**	29 (50.5%)	26 (49.1%)	30 (50.8%)		
**Mean time (s) after fluorescein dye injection when counting microangiomas**	30.24 ± 2.41	29.51 ± 2.95	30.43 ± 3.06	1.632	0.199
**Mean time (s) after fluorescein dye injection during calculation of retinal neovascular area**	40.10 ± 3.94	40.53 ± 3.90	40.97 ± 3.59	0.761	0.468

PRP, retinal laser photocoagulation; O. D, right eye; L. E, left eye.

#### 2.1.2 Diagnostic criteria for high-risk PDR

The concept of high-risk PDR emerged in 1991 and was proposed by the early treatment diabetic retinopathy study (ETDRS) research group ^[104]^. According to DR treatment guidelines in China, high-risk PDR is defined as NV of the disc of > 1/4–1/3 disc diameter (DD) or NV elsewhere in the retina of > 1/2 DD, accompanied by pre-retinal or vitreous hemorrhage. High-risk PDR should be treated timely with PRP ^[105]^. This study used the definition of high-risk PDR in the 2014 Chinese guidelines for clinical diagnosis and treatment of DR.

#### 2.1.3 Inclusion criteria

The inclusion criteria included: patients with DR at high risk of PDR; patients whose OCT showed a central retinal thickness (CRT) of 250 μm or a best-corrected visual acuity (BCVA) 25~73 ETDRS letters; no vitreous hemorrhage, retinal detachment, and other surgical indications; and patients compliant with the treatment follow-up plan.

#### 2.1.4 Exclusion criteria

The exclusion criteria included: DR caused by age-related macular degeneration and retinal vein occlusion; history of using other anti-VEGF drugs within 3 months; patients with a history of laser treatment within 6 months, affecting macular function; history of intraocular hormone therapy within 6 months; history of vitreoretinal surgery; patients with unclear refractive media affecting the measurement results; and patients with poor general condition.

### 2.2 Study methods

#### 2.2.1 Data collection

**Figure 1:**
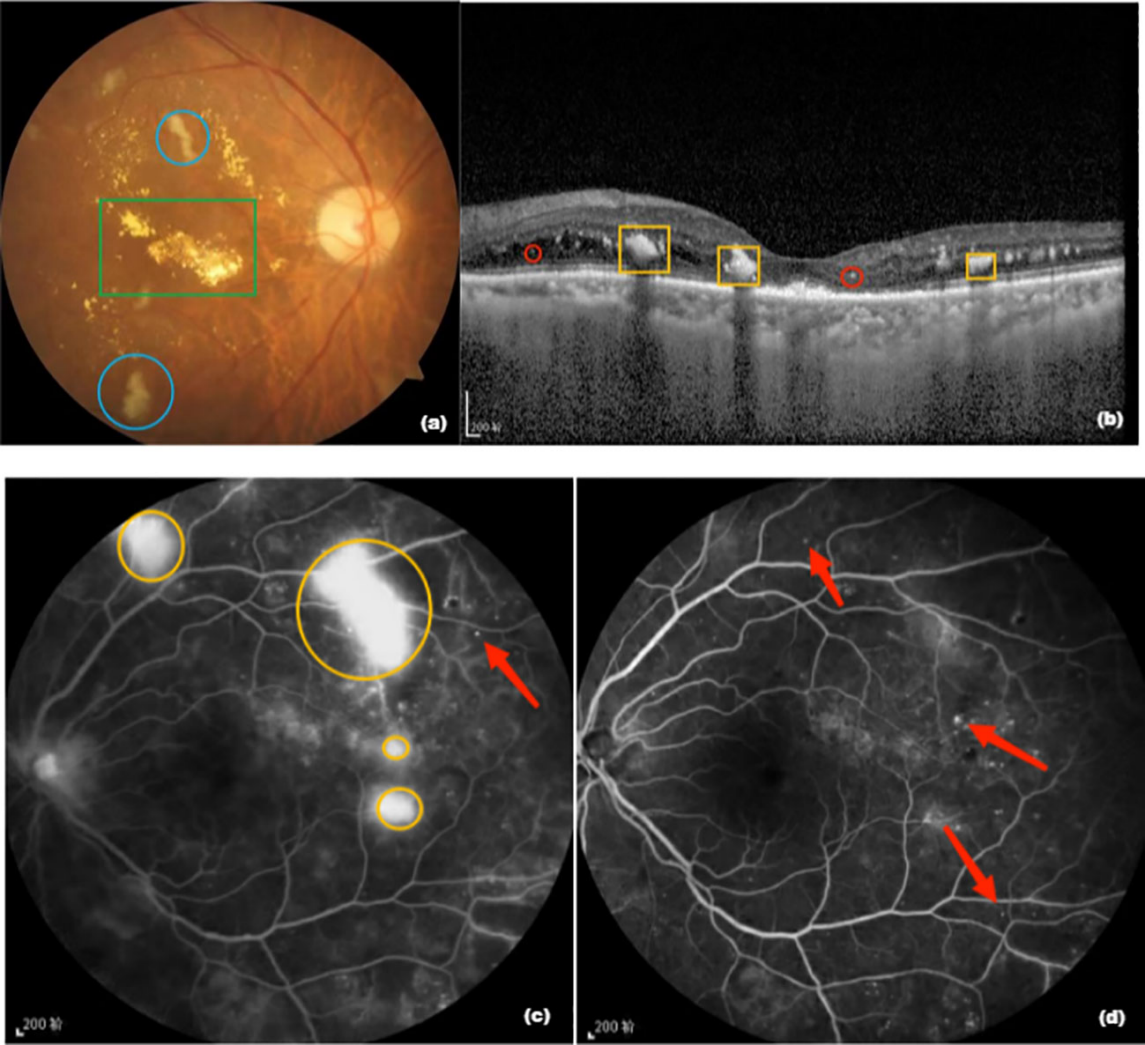
**(A)** Fundus photograph. The green box is a hard exudate, yellowish-white, and the area shown in blue circle is an absorbent cotton spot, also called “soft exudate”; **(B)** Optical coherence tomography image. The yellow box is a hard exudate and the structure shown in red circle is a highly reflective point; **(C)** Fundus fluorescence image before treatment. The yellow circle is the neovascularization area and the red arrow points to the microvascular tumor; **(D)** Fluorescence imaging image of the fundus of the patient after 12 months of aflibercept treatment showing that neovascularization has disappeared and the microvascular tumor is still partially present.

Data on the medical history and examination results of the patients were obtained from the hospital’s electronic case management system, and relevant follow-up indicators were obtained by telephone. At the monthly follow-up, all patients were examined using BCVA, intraocular pressure, fundus photography, OCT, and fundus fluorography. BCVA was observed using the EDTRS visual chart and converted into LogMAR. CRT was measured using OCT (model Cirrus HD-OCT 5000, Carl Zeiss) and hard exudation (HE) area was observed using VISUCAM224 fundus camera. Data processing analysis was performed. CRT was defined as the average of three measurements of the mean thickness from the inner to the outer central 1 mm of retinal tomography, measured by the same experienced technician. A boundary line between the HE area and NV vessels was drawn from the edge of the optic disc to the temporal 2PD of all HE areas and NV vessels to capture the area of each HE and NV vessel, which were added to obtain the total area. Observations in the peripheral retina were excluded to unify the results before and after examination within and between the patient groups. All results were reviewed by professional ophthalmologists (**Figure 1**).

Macular area (MA) and hemorrhage were distinguished by comparing the results of color fundus photography and fundus fluorescein angiography. Fundus fluorescence imaging (FFA) was performed using a Spectralis HRA laser device, aimed at the patient’s eye. Fluorescein sodium diluent (3 mL) was injected into the antecubital vein and the patient was observed for any discomfort 10 min later. Patients that showed no adverse events were administered 5 mL of 20% fluorescein sodium diluent, and the retina of the optic disc, posterior pole, and MA were captured and recaptured after 10 min, 15 min, and 20 min using the same shooting range, and all photos were archived.

In addition, adverse events during treatment were recorded. Four patients in the PRP group discontinued treatment owing to massive vitreous accumulation and were switched to treatment by vitrectomy, while those receiving aflibercept and PRP were tested as expected.

#### 2.2.2 Treatment methods

##### 2.2.2.1 Pre-operative laser preparation

The naked eye, corrected far and near vision, intraocular pressure, cornea, pupil, anterior chamber, iris, and lens were checked, and color fundus image and fundus fluorescein angiography were performed. Patients and their family members were informed that the laser therapy was designed to enhance or improve existing vision and reduce the risk of deterioration, which may cause visual fluctuations and mild eye pain during processing, and that there will be repeated postoperative and photocoagulation examinations. Patients were required to sign an informed consent form before the operation. Except for angle-closure glaucoma, pupil dilation drugs were used to widen the pupil. Furthermore, contact lenses were cleaned and disinfected. The laser equipment was checked. Generally, argon ion, argon green, or argon blue-green lasers were used more frequently. The patient was positioned appropriately, and surface anesthesia was applied to the eye. Whole PRP was performed according to the scheme recommended by ETDRS, with the external optic disc connected to the equator, 2 DD from the macular center and temporal side, and the disseminated laser photocoagulation outside the formed oval range. Photocoagulation was performed on the nasal, lower, upper, and temporal sides, and the interval between each photocoagulation was 1 week. The size of the light dots was 200–500 microns, and the energy consumption was 200–300 uW over an exposure time of 0.2 – 0.3 s. The number of spots in this treatment was not more than 500, and the diameter of the two rooms was 1–1.5 spots. During the follow-up procedure, PRP salvage therapy was allowed. In addition, the patients receiving PRP with aflibercept received a vitreous injection of aflibercept before PRP, which effectively prevented macular swelling caused by short-term laser photocoagulation and the interruption of vitreous hemorrhage treatment. Changes in intraocular pressure and the presence of vitreous hemorrhage were monitored.

Intravitreal injection and aqueous humor collection were performed in the ophthalmic operating room. Before administering the injection, the conjunctiva sac and lacrimal duct were cleaned, disinfected, and covered. Surface anesthesia was applied to the patient’s eye in the conjunctiva sac. Aqueous humor samples were collected from all patients and rinsed continuously before treatment with aflibercept intraocular injection.

##### 2.2.2.2 Operation procedure

All patients had their aqueous humor collected before treatment with the intraocular injection. After surface anesthesia was applied to the affected eye, the operator opened the eyelid and used an insulin needle to collect 100 μL of aqueous humor in the anterior chamber, 1.0 mm of the limbal ring. Immediately after collection, the aqueous humor was placed in a sterile Eppendorf tube and kept in the refrigerator at -80°C for further tests.

Intravitreal injection of 2 mg aflibercept was administered into the angular scleral margin with a spacing of 3.5 mm. After the injection, the needle was gently pressed with a cotton swab for approximately 10–15 s. After observing the eye, tobramycin-dexamethasone eye ointment was applied and covered with gauze. The patients in the 5 + PRN and 3 + PRN groups initially received 5 and 3 monthly 2 mg intravitreal aflibercept injections, respectively, according to the following conditions: 5 ETDRS letters decrease in BCVA and 100 μ m increase in CRT. During the initial treatment period, follow-up was performed once a month, followed by once every other month for 1 year.

#### 2.2.3 Sample testing

At baseline and 12 months of follow-up, the aqueous humor was extracted for testing. During the study, the immediate segment, intraocular pressure, and fundus were carefully observed, and changes were recorded. During the follow-up, the equipment and methods used before and after treatment were the same for BCVA, intraocular pressure, fundus photography, OCT, and FFA measurements, which were performed by three professional eye technicians.

The aqueous humor was tested using the flow-through liquid-phase multiplex protein quantification technique to determine VEGF and monocyte chemoattractant protein-1(MCP-1) content. The operation was as follows: aqueous humor samples were centrifuged at 4°C and 1000 g for 15 min. Subsequently, 50 uL of MCP-1 supernatant was obtained and tested for VEGF. Next, 45 μL capture bead diluent was injected into a 1.5 ml centrifugation tube, and MCP-1 and VEGF beads were violently shaken using the oscillator for 15 s. Thereafter, 1 μL of the solution was mixed with 45 μL of bead diluent and finally mixed with shock. Next, 50 uL of aqueous humor was added to the solution, fully mixed using the oscillometer, and finally placed in a dark environment for 1 h. In a 1.5 ml centrifugation tube, 45 μ ml of detection reagent diluent solution, hand-flicked MCP-1 PE, and VEGF PE, were mixed with gentle shaking. The configured PE solution was placed in a dark centrifugal pipe in the second step for 2 h, and 1 ml BD wash buffer was added to the completely covered and clean centrifugal tube. The cap of the centrifugal tube was poured two times, and centrifuged at 200 g. The liquid was discharged, and 300 micro LBD aqueous solution was added. The centrifugal tube was covered and rotated with a finger to suspend the beads completely and placed into the flow falcon injection tube. After cleaning and starting the software, the software was operated by inserting the injection needle, setting the parameters, and reading the data. The value of each index was calculated using the standard curve expression rather than reading. Glial fibrillary acidic protein (GFAP) content of the aqueous humor was determined by the enzyme-linked immunosorbent assay (ELISA) (1). Detection protein and the corresponding buffer agent were selected. On the day of the test, the aqueous humor samples were removed from the refrigerated room of -80^0^C into the refrigerated room of 4^0^C for thawing, to determine the number of samples and verify the identity of the experimenter. (2) A standard solution was made by adding 20 μl of aqueous humor to 380 microliters of diluent. We made 3000 pg/ml of the top standard solution and repeated it twice to obtain seven gradually diluted standard solutions. For sample preparation, 50 μl of aqueous humor was required for each sample, which was added to the microplate and incubated with 50 μl of antibody for 1 h. The microplate was washed with the washing liquid thrice after incubation, and 100 μl of TMB was added to the microplate and incubated for 10 min. Thereafter, 100 μl of the termination solution was added (8). Using the microplate reader set at 450 nm, OD was determined. The standard curve was compared with that of the OD and OD of the standard, and the sample OD was introduced into the standard curve to obtain the GFAP content of the sample.

### 2.3 Statistical methods

Statistical analysis was performed using SPSS software version 23.0 (Armonk, NY: IBM Corp). The χ^2^ test was used to compare count data, analysis of variance was used for statistical description of measurement data, Student’s *t*-test was used to analyze independent samples, and the Student–Newman–Keuls test was used for group comparisons. A P-value of less than 0.05 was considered statistically significant.

## 3 Results

### 3.1 Changes in the BCVA before and after treatment

Post-treatment, the BCVA (log MAR) increased slightly in the PRP group from 0.69 ± 0.17 to 0.71 ± 0.17, with no significant difference (P>0.05). However, in the aflibercept 5 + PRN with PRP and aflibercept 3 + PRN with PRP groups, the BCVA (log MAR) decreased from 0.72 ± 0.17 and 0.74 ± 0.17 to 0.50 ± 0.13 and 0.53 ± 0.17, respectively, with statistically significant differences (all P<0.05). Statistically significant differences were observed between the aflibercept 5 + PRN with PRP and aflibercept 3 + PRN with PRP groups and PRP alone group (Q=9.794, P<0.05; Q=8.388, P<0.05, respectively). However, no significant difference was observed between the two combined treatment groups (Q=1.638, P>0.05) (**Table 2**).

**Table 2:** Changes in the BCVA (LogMAR) before and after treatment.

	Pretherapy BCVA (log MAR)	BCVA (log MAR) after 12 months of treatment	T	P
**PRP group (n=58)**	0.69 ± 0.17	0.71 ± 0.17	0.440	0.661
**Aflibercept 5 + PRN combination group** **(n=53)**	0.72 ± 0.17	0.50 ± 0.13	7.514	0.000
**Aflibercept 3 + PRN combined group** **(n=59)**	0.74 ± 0.17	0.53 ± 0.17	6.795	0.000
**F**	1.203	28.210		
**P**	0.305	0.000		

Q =9.794, P <0.05 for PRP-only; Q =8.388, P <0.05 for PRP vs. aflibercept 3 + PRN with PRP and PRP vs. aflibercept 5 + PRN with PRP. Q =1.638 for pairwise comparison of the three samples (Newman–Keuls method), BCVA: best-corrected visual acuity.

### 3.2 Changes in central macular foveal thickness before and after treatment

Central macular foveal thickness (CFT) slightly decreased in the PRP group from 361.80 ± 36.70 μm to 353.86 ± 40.88 μm, with no significant difference (P>0.05). In the aflibercept 5 + PRN with PRP and aflibercept 3 + PRN with PRP groups, CFT decreased from 356.57 ± 37.57 μ m and 358.17 ± 44.66 μ m to 284.87 ± 31.52 μ m and 303.19 ± 37.00 μ m, respectively, with statistically significant differences observed before and after treatment (P<0.05 in both groups). There were significant differences between the three groups after treatment (Q=13.947, P<0.05 for PRP vs. aflibercept 5 + PRN and PRP alone; Q=10.528, P<0.05 for aflibercept 3 + PRN and PRP vs. aflibercept 5 + PRN with PRP; Q=3.718, P<0.05). In the aflibercept 5 + PRN with PRP group, the decrease in CFT was significantly better than that in the aflibercept 3 + PRN with PRP and PRP-only groups (**Table 3**).

**Table 3:** Changes in the CFT before and after treatment.

	Pre-treatment CFT (μ m)	CFT after treatment (μ m)	T	P
**PRP group (n=58)**	361.80 ± 36.70	353.86 ± 40.88	1.110	0.270
**Aflibercept 5 + PRN combined group** **(n=53)**	356.57 ± 37.57	284.87 ± 31.52	10.645	0.000
**Aflibercept 3 + PRN combined group** **(n=59)**	358.17 ± 44.66	303.19 ± 37.00	7.282	0.000
**F**	0.261	53.134		
**P**	0.771	0.000		

Q =13.947, P <0.05 for PRP vs. aflibercept 5 + PRN with PRP; Q =10.528, P <0.05 for aflibercept 3 + PRN with PRP vs. aflibercept 5 + PRN with PRP, P <0.05; q-wise comparison of three samples (Newman–Keuls method); CFT, central foveal thickness.

### 3.3 Changes in the number of MA before and after treatment

The number of MA (one) in the PRP, aflibercept 5 + PRN with PRP, and aflibercept 3 + PRN with PRP groups decreased from 118.34 ± 27.96, 118.60 ± 33.34, and 116.59 ± 28.95 to 92.95 ± 29.04, 44.60 ± 20.73, and 54.26 ± 25.43, respectively (P<0.05 in all groups). For the three pairwise comparisons, the differences were all statistically significant (Q=14.156, P<0.05 for PRP vs. aflibercept 5 + PRN; Q=11.643, P<0.05 for aflibercept 3 + PRN with PRP vs. aflibercept 5 + PRN with PRP). In the aflibercept 5 + PRN with PRP, the decrease in the number of MA was significantly better than that in the aflibercept 3 + PRN with PRP and PRP groups (**Table 4**).

**Table 4:** Changes in MA before and after treatment.

	Number of MA before treatment	MA number after 12 months of treatment	T	P
**PRP group (n=58)**	118.34 ± 27.96	92.95± 29.04	4.797	0.000
**Aflibercept 5 + PRN with PRP group (n=53)**	118.60 ± 33.34	44.60 ± 20.73	13.102	0.000
**Aflibercept 3 + PRN with PRP group (n=59)**	116.59 ± 28.95	54.26 ± 25.43	12 426	0.000
**F**	0.084	57.361		
**P**	0.927	0.000		

Q =14.156, P <0.05 for PRP vs. aflibercept 5 + PRN with PRP; P <0.05 for PRP versus PRP; Q =11.643 for aflibercept 3 + PRN with PRP vs. aflibercept 5 + PRN with PRP, P <0.05; q-test (Newman–Keuls method), MA, Microhemangioma.

### 3.4 Changes in the retinal NV area before and after treatment

The retinal NV area in the PRP, aflibercept 5+PRN with PRP, and aflibercept 3+PRN with PRP groups decreased from 1.00 ± 0.21 mm², 1.01 ± 0.18 mm², and 0.98 ± 0.20 mm² to 0.49 ± 0.17 mm², 0.31 ± 0.16 mm², and 0.38 ± 0.14 mm², respectively. All the differences were statistically significant before and after treatment (P<0.05 in all groups). Pairwise comparisons showed statistically significant differences between the PRP and aflibercept 5 + PRN with PRP groups and between the PRP and aflibercept 3 + PRN with PRP groups (Q=8.627, P<0.05 and Q=5.725, P<0.05, respectively). However, no significant difference was observed between aflibercept 5 + PRN with PRP and aflibercept 3 + PRN with PRP groups (Q=3.068, P>0.05) (**Table 5**).

**Table 5:** Changes in NV area before and after treatment.

	Pretherapy New vessel area	After 12 months of treatment New vessel area	T	P
**PRP group (n=58)**	1.00 ± 0.21	0.49 ± 0.17	14.359	0.000
**Aflibercept 5 + PRN with PRP group (n=53)**	1.01 ± 0.18	0.31 ± 0.16	27.866	0.000
**Aflibercept 3 + PRN with PRP group (n=59)**	0.98 ± 0.20	0.38 ± 0.14	19.415	0.000
**F**	0.121	19.340		
**P**	0.891	0.000		

Q =8.627, P <0.05 for PRP vs. aflibercept 5 + PRN with PRP; Q =5.725 for aflibercept 3 + PRP with PRP, P> 0.05; the q-wise comparison of three samples (Newman–Keuls method), NV: neovascularization; PRP, retinal laser photocoagulation.

### 3.5 Changes in the HE area before and after treatment

After 12 months of treatment, in the PRP group, patients without HE increased from two to nine, patients with an HE area of less than 0.5 mm² increased from 23 to 29, patients with an HE area of 0.5 mm²–2.5 mm² reduced from 22 to 13, and patients with an HE area of more than 2.5 mm² decreased from 11 to seven. In the 5 + PRN with PRP group, patients with no HE area increased from two to 20. The number of patients with an HE area of less than 0.5 mm² remained unchanged, patients with an HE area of 0.5 mm²–2.5 mm² reduced from 20 to eight, and patients with an HE area of more than 2.5 mm² reduced from eight to two. Patients without HE before treatment In the 3 + PRN with PRP group increased from three to 21, patients with an HE area of less than 0.5 mm² decreased from 25 to 19, patients with an HE area of 0.5 mm²–2.5 mm² decreased from 21 to 16, and patients with an HE area of more than 2.5 mm² reduced from 10 to three. The differences were all statistically significant (χ*^2 = ^* 8.3500, 23.4701, and 18.7631, respectively; P<0.05) (**Table 6**).

**Table 6:** Changes in HE areas before and after treatment.

	HE area	Before therapy	At 12 months after the treatment	x^2^	P
**PRP group (n=58)**	no HE	2	9	8.350	0.039
	<0.5 mm²	23	29		
	0.5 mm²–2.5 mm²	22	13		
	>2.5 mm²	11	7		
**Aflibercept 5 + PRN with PRP group (n=53)**	no HE	2	20	23.470	0.000
	<0.5 mm^2^	23	23		
	0.5 mm²–2.5 mm²	20	8		
	>2.5 mm²	8	2		
**Aflibercept 3 + PRN with PRP group (n=59)**	no HE	3	21	18.763	0.000
	<0.5 mm^2^	25	19		
	0.5 mm²–2.5 mm²	21	16		
	>2.5 mm²	10	3		
**x^2^**		0.610	13.160		
**P**		0.996	0.041		

HE, hard exudate area; PRP, retinal laser photocoagulation.

### 3.6 Changes in VEGF levels in the aqueous humor before and after treatment

VEGF in the aflibercept 3 + PRN with PRP group and aflibercept 5 + PRN with PRP decreased from 156.33 ± 11.30 pg/mL and 154.46 ± 9.67 pg/mL to 8.81 ± 4.28 pg/mL and 8.44 ± 4.85 pg/mL, respectively (P<0.05 in both groups). After treatment, no statistical difference was observed between aflibercept 5 + PRN with PRP and aflibercept 3 + PRN with PRP (P>0.05) (**Table 7**).

**Table 7:** Changes in VEGF levels in the atrial fluid before and after treatment.

	Pre-treatment (pg/mL)	After 12 months of treatment (pg/mL)	T	P
**Aflibercept 3 + PRN with PRP group (n=59)**	156.33 ± 11.30	8.81 ± 4.28	95.780	0.000
**Aflibercept 5 + PRN with PRP group (n=53)**	154.46 ± 9.67	8.44 ± 4.85	93.965	0.000
**t**	0.935	0.430		
**P**	0.352	0.668		

Comparison of changes in VEGF levels in the aqueous humor before and after treatment using the t-test. VEGF: vascular endothelial growth factor; PRP, retinal laser photocoagulation.

### 3.7 Changes in the levels of MCP-1 in the aqueous humor before and after treatment

MCP-1 levels in the aflibercept 3 + PRN with PRP and aflibercept 5 + PRN with PRP groups significantly increased from 978.05 ± 96.93 pg/mL and 997.00 ± pg/mL to 237.57 ± 93.31 and 2222.78 ± 86.79 pg/mL, respectively (P<0.05 in both groups). However, no significant difference was observed between the MCP-1 level in the aflibercept 5 + PRN with PRP and aflibercept 3 + PRN with PRP groups (P>0.05) (**Table 8**).

**Table 8:** Changes in monocyte chemotactic protein (MCP-1) levels in the atrial fluid before and after treatment.

	Pre-treatment (pg/mL)	After 12 months of treatment (pg/mL)	T	P
**Aflibercept 3 + PRN with PRP group (n=59)**	978.05 ± 96.93	237.57 ± 93.31	42.841	0.000
**Aflibercept 5 + PRN with PRP group (n=53)**	997.00 ± 108.74	222.78 ± 86.79	47.319	0.000
**T**	0.975	0.866		
**P**	0.332	0.389		

Changes in MCP-1 levels in aqueous humor before and after treatment were compared using the t-test. MCP-1: monocyte chemoattractant protein; retinal laser photocoagulation.

### 3.8 Changes in the level of GFAP in the aqueous humor before and after treatment

GFAP levels in the aflibercept 3 + PRN with PRP and aflibercept 5 + PRN with PRP groups significantly reduced from 1.39 ± 0.20 (t=0.18) and 0.26 ± 0.18) pg/mL to 0.26 ± 0.11 and 0.28 ± 0.13, respectively (P<0.05 in both groups). However, no significant difference was observed in GFAP levels between aflibercept 5 + PRN with PRP and aflibercept 3 + PRN with PRP groups (P>0.05) (**Table 9**).

**Table 9:** Changes in glial fibrillary acidic protein (GFAP) levels in the atrial fluid before and after treatment.

	Pre-treatment (pg/mL)	After 12 months of treatment (pg/mL)	T	P
**Aflibercept 3 + PRN with PRP group (n=59)**	1.39 ± 0.20	0.26 ± 0.11	39.403	0.000
**Aflibercept 5 + PRN with PRP group (n=53)**	1.32 ± 0.18	0.28 ± 0.13	36.418	0.000
**T**	1.961	0.818		
**P**	0.052	0.415		

Comparison of changes in GFAP levels in the aqueous humor before and after treatment was performed using the t-test. GFAP: glial fibrillary acidic protein; PRP, retinal laser photocoagulation.

## 4 Discussion

### 4.1 Research background

In the early 1990s, through extensive study of the mechanisms underlying DR, a classification of DR based on severity was proposed which is still widely used. In the absence of NV, DR is classified as non-proliferative (NPDR), ranging from mild to severe. In the presence of NV, DR is classified as proliferative (PDR). The risk of progression to advanced PDR is highly dependent on baseline disease levels; compared with severe NPDR, moderate NPDR progresses to PDR after 1 year, with a 12–27% risk and a 52% risk of PDR over the same period (11). By analyzing the mechanism underlying DR, four factors affecting DR were identified. The patient’s vision is severely impaired within 2 years (defined as a 5/200 diagnosis of no less than twice every 5 months) (12). As the number of risk factors increased from two to three, the risk of severe visual loss increased from 8.5% to 26.7%.

In patients with diabetes, impaired retinal pigment epithelium pumping capacity and choroidal blood flow limit the migration of water and lipids from the outer retinal layer to the choroid. Thus, HE’s are formed, expanded, and deposited, and are more likely to occur in the macular fovea, accompanied by a rapid decrease in CFT in patients with macular edema. Several additional studies reported that diabetic macular edema (DME) with subretinal fluid (SRF) may be associated with the formation of hyper-reflective foci that may precede and extravasate the deposition of plasma lipids and/or proteins comprising HE. The longer the duration of cystoid macular edema, the higher the risk of HE deposition in the macular region during follow-up. We speculate that long-term macular edema may increase the concentration of inflammatory cytokines and VEGF in the vitreous fluid, which is significantly associated with the presence of SRF (13), whereas more exudates were deposited within the macular retina to form a clinical HE. However, there is still controversy about whether the origin of the hyper-reflective foci is a precursor of HE (14, 15). Therefore, the relationship between SRF and HE deposition in the macula must be further explored.

Cytokines are small molecule proteins with strong biological functions. Studies have shown that cytokines can be divided into interleukins (ILs), interferons, tumor necrosis factor superfamily, colony-stimulating factors, chemokines, and growth factors. Cytokines are closely associated with various ocular diseases, such as DME, neovascular glaucoma, DR, and other immune diseases. A validation experiment using ELISA showed that ANG-1, ANG-2, IL-6, VEGF, MMP-9, hepatocyte growth factor (HGF), placental growth factor (PIGF), depolin and metalloproteinase (ADAM) -11, chemokines (CXCL) -10, IL-8, and platelet-derived growth factor (PDGF) -A were higher than the control group (16). However, few clinical studies exist on the relationship between cytokines, such as MCP-1 and GFAP, and disease progression in patients with PDR and the changes after anti-VEGF treatment. We collected the concentrations of VEGF, MCP-1, GF, and GFAP in the aqueous humor before and after treatment, observed the changes in the cytokine level after PRP and aflibercept with PRP treatment, and explored the prognostic relationship among patients with high-risk PDR.

We found that some patients with good vision already fulfilled the criteria for high-risk PDR at the first visit, and the new blood vessels appearing in the inner retina had ruptured, resulting in vitreous hemorrhage, and severe decline in vision. To reduce this risk, PRP must be administered strictly as prescribed. Anti-VEGF drugs are now used in clinical practice. However, based on the economic situation in some poor areas in China, some patients choose PRP treatment alone because they cannot afford the cost of anti-VEGF drugs.

Among various anti-VEGF drugs, aflibercept is an emerging drug, and its effect has been fully confirmed after phase VIVID and VISTA III clinical trials (17). Chen Zhen, a Chinese medical expert (18) and Lu Qianyi (19) have verified its efficacy. However, the previous research was conducted among Caucasians. Domestically, research knowledge is limited to the short-term treatment of DME for 1 or 2 months, and its long-term effects have not been elucidated.

Therefore, this study used PRP alone, aflibercept 3 + PRN and PRP, and followed expert consensus (20). Aflibercept 5 + PRN with PRP regimen was recommended for patients with both high-risk PDR and DME. At baseline and after 12 months of treatment, BCVA (Log MAR), CFT, MA, NV area, and HE changed. The treatment regimen of the combined treatment group comprised an intravitreal injection before performing PRP. The reasons were as follows (1): compared with the half-life of anti-VEGF drugs, such as ranezumab and comaccept, the intravitreal half-life of aflibercept is usually more than 10 days (21); (2) macular edema caused by retinal laser photocoagulation mostly occurs during photocoagulation or 1 month after treatment (22). Thus, an intravitreal aflibercept injection was administered 1 week before PRP.

### 4.2 Factors influencing the efficacy of anti-VEGF therapy in DR treatment

We found that the BCVA was better in the PRP group. The BCVA (log MAR) in the PRP alone group did not improve after treatment; however, it increased from 0.69 ± 0.17 to 0.71 ± 0.17, indicating that PRP alone could not delay or prevent further decline in visual acuity. This is also consistent with previous studies. Rebecca et al. (23) reported that after PRP, patients showed lower BCVA in the 1st and 3rd months compared with that before PRP. Moreover, Zhao et al. (24) showed that the incidence of BCVA was significantly decreased at 1 and 3 months after PRP compared with that before PRP in patients with DR. Lorusso et al. (25), reported lower BCVA than the basal levels 6 months after PRP in patients with DR.

In this study, intravitreal aflibercept injection had no significant effect on the NV and HE area (P>0.05); hence, we speculated that it may be related to the distribution of HE and NV in different locations in the retina of the patients. In 2020, Li Xiaoli et al. (26) analyzed the distribution of patients with DR using FFA and color fundus photography FP and found that HE occurs mostly in the posterior pole (69.7%). In severe NPDR and hyperplastic DR, intraretinal microvascular abnormality, NPA, and NV were more common on the nasal side, especially in the inferior nasal region (60.3%, 38.7%, and 76.0%, respectively). Although our study participants were all patients with high-risk PDR, their blood glucose control and disease progress were not used to classify the location of HE and NV within the groups, which may have led to inconsistent responses to anti-VEGF drugs and, thus, affected the prognosis of retinal anatomical structure and function.

In addition, we found that the concentration of cytokines, VEGF, MCP-1, and GFAP in the aqueous humor improved after treatment compared with that before treatment; however, no statistical difference was observed in the aflibercept 5 + PRN group (P>0.05). This may have been due to the patient’s genotype, blood pressure level, blood sugar level, blood lipid level, renal function, smoking history, insomnia, and the interaction between cytokines affecting the expression of Muller cells. The reasons are given as follows:

Several studies have shown that smoking duration and blood pressure can affect the concentration of MCP-1 in patients. Komiyama et al. (27), in a large sample analysis of serum MCP-1 concentrations, after adjusting for sex differences, found that serum MCP-1 concentration was positively associated with smoking duration in smokers and that smoking duration was an independent determinant of serum MCP-1 concentration in smokers. They also found that in smokers, serum MCP-1 concentrations were significantly higher than that in normotensive patients, systolic blood pressure was an independent determinant of serum MCP-1 concentration in smokers, and ACS was more likely to occur in long-term smokers. Unlike in smokers, serum MCP-1 concentration was not associated with systolic or diastolic blood pressure. The study found that the serum MCP-1 concentration in patients with hypertension was significantly higher than that in patients without hypertension. MCP-1 concentration gradually increased with increased hypertension grade, and the gap between the levels was statistically significant (P<0.05). Notably, MCP-1 concentration is related to genotype. A domestic study showed that MCP-1 c.2518G/G is a susceptibility gene for DR, especially high-risk PDR among patients with type 2 diabetes in the Han population in Northern China. The G allele was associated with DR severity in the A/G polymorphism, while the G allele showed no obvious correlation with DR, requiring further studies (28).

In addition to MCP-1, VEGF expression is associated with genotype. Increasing evidence suggests that miR-126 is involved in the regulation of genes closely related to angiogenesis and inflammation. Studies have detected significantly high expressions of miR-126 in the vitreous tissue, proliferative membrane tissues, and the plasma of the affected eyes of patients with PDR compared with patients without PDR. Furthermore, miR-126 expression was significantly higher in the vitreous and proliferation membrane tissues in patients with stage VI DR than in patients with stage V and IV DR. In other words, miR-126 expression increased with increasing PDR severity. It is suggested that miR-126 is abnormally highly expressed in patients with PDR and may be a potential mechanism underlying PDR development (29).

Since there are two large glial cell populations in the retina, namely the astrocytes and Muller cells, which can all express cytokines, such as VEGF, GFAP, and MCP-1, we cannot determine the source of cytokines in the aqueous humor. Alternatively, the expression of other inflammatory cytokines may have an impact on the function of Muller cells. Previous studies have demonstrated that IL-17A promotes functional impairment in the Muller cells through Act 1 signaling. Th 22 cells are involved in DR pathogenesis by directly promoting retinal Muller-cell activation and dysfunction as well as BRB disruption and inflammatory response via IL-22 production. In addition, Th 22 cells can secrete IL-22, IL-22, and IL-22 R α 1 to activate the Act 1/TRAF 6 pathway and promote an inflammatory response in Muller cells (30). However, whether they could further affect the expression of VEGF, MCP-1, and GFAP by Muller cells is unknown and needs to be investigated.

In this study, the differences in CFT and MA number between the aflibercept 5 + PRN with PRP and aflibercept 3+PRN with PRP groups were significant before and after treatment (P<0.05, in all groups); the efficacy of aflibercept 5 + PRN with PRP was significantly better than PRP alone and aflibercept 3 + PRN with PRP. This suggests that increasing the aflibercept loading dose can significantly reduce CFT and MA for the same treatment time. We speculated that by increasing the loading dose, we could significantly improve vascular permeability, reduce inflammation and oxidative stress, reduce the generation of microhemangiomas, reduce retinal ischemia and hypoxia, increase blood flow, and reduce vascular leakage, which can help DR regress at an earlier date. Therefore, although there was no statistically significant difference between aflibercept 5+PRN with PRP and aflibercept 3+PRN with PRP in BCVA after 12 months of treatment, in the long run, it may reduce the destruction of the retinal structure and visual function in patients with DR. Therefore the aflibercept 5+PRN with PRP regimen is necessary for patients with DR.

As for BCVA, NV, and HE area, and whether the observation indicators, such as cytokines in the aqueous humor, will predict a better prognosis based on our treatment strategy and follow-up period, further investigation is needed to clarify the role of MA, HE, genotype, smoking history, sleep quality, blood pressure level, blood sugar level, blood lipid level, and hemoglobin level through long-term observation and detailed statistical analysis.

### 4.3 Safety of intravitreal aflibercept injection

In this study, no serious adverse events occurred except that a few patients experienced subconjunctival hemorrhage and transient elevated intraocular pressure after intravitreal injection. Intravitreal aflibercept administration is safe and well tolerated, with a few side effects; however, strict clinical evaluation of poor systemic conditions, such as advanced age, patients with a recent history of cardiovascular and cerebrovascular disease, and poor blood glucose control, should be performed before intravitreal aflibercept administration.

### 4.4 Strengths and limitations of the study

The main objective of this study was to observe the effectiveness and safety of different dosage regimens of intravitreal aflibercept injection with PRP in patients with high-risk PDR and DME. Although the patients chose the treatment plan independently, their choice may be somewhat related to their economic level, living standard, and other factors. However, the baseline data of the patients in the three groups were not significantly different, except for the number of injections they received (P>0.05), indicating that the three groups were comparable, and selection bias was reduced to some extent. In addition, based on previous studies on anti-VEGF treatment after MCP-1, GFAP, and the relationship between the concentration and DR, previous animal studies showed the occurrence and development of DR; therefore, we hope that we can observe the severity of DR, as a clinical prediction of patient prognosis index in the future. However, this study had some limitations. First, the general condition of the patients, such as genotype, body mass index, blood pressure level, blood glucose level, blood lipid level, microalbumin level, smoking history, and sleep status, were not considered nor evaluated. These factors may affect the prognosis of the disease. Future studies should consider these factors when performing data collection and analysis. Second, NV and HE areas were all measured manually, which is prone to inevitable statistical errors. Developing automatic measurement tools, such as through artificial intelligence, is required to provide a higher detection accuracy. Third, only three cytokines, VEGF, MCP-1, and GFAP, were observed, and the levels of other inflammatory cytokines and chemokines, which may have an effect on the expression of Muller cells, were not monitored. Finally, the long-term effect and safety of multiple aflibercept injections was not determined.

In conclusion, aflibercept 5 + PRN intravitreal injection with PRP was safe and effective in treating patients with both PDR and DME, and it improved CFT better than in 3 + PRN (MA); however, BCVA, NV, and HE area, and VEGF, MCP-1, GFAP cytokines in aqueous aflibercept 3 + PRN group were not improved. CFT and MA can be used as early indicators for observing the improvement and regression of DR; however, their long-term clinical efficacy needs further investigation.

## Data availability statement

The original contributions presented in the study are included in the article/supplementary material. Further inquiries can be directed to the corresponding authors.

## Author contributions

SL: Writing – original draft. YT: Formal analysis, Writing – review & editing. SY: Data curation, Writing – review & editing. MY: Resources, Writing – review & editing. HZ: Formal analysis, Writing – review & editing. MS: Investigation, Writing – review & editing. WC: Resources, Writing – review & editing. HW: Supervision, Writing – review & editing.

## References

[1] Yeh HC Brown TT Maruthur N Ranasinghe P Berger Z Suh YD. Comparative effectiveness and safety of methods of insulin delivery and glucose monitoring for diabetes mellitus: a systematic review and meta-analysis. Ann Intern Med (2012) 157:336–47. doi: 10.7326/0003-4819-157-5-201209040-00508 22777524

[2] Wong TY Sabanayagam C. The war on diabetic retinopathy: where are we now? Asia Pac J Ophthalmol (Phila) (2019) 8:448–56. doi: 10.1097/APO.0000000000000267 PMC6903323 31789647

[3] Chen XD Gardner TW. A critical review: psychophysical assessments of diabetic retinopathy. Surv Ophthalmol (2021) 66:213–30. doi: 10.1016/j.survophthal.2020.08.003 PMC7914308 32866468

[4] Ruta LM Magliano DJ Lemesurier R Taylor HR Zimmet PZ Shaw JE. Prevalence of diabetic retinopathy in Type 2 diabetes in developing and developed countries. Diabetes Med (2013) 30:387–98. doi: 10.1111/dme.12119 23331210

[5] Giuliari GP Sadaka A Chang PY Cortez RT. Diabetic papillopathy: current and new treatment options. Curr Diabetes Rev (2011) 7:171–5. doi: 10.2174/157339911795843122 21418004

[6] Masser DR Otalora L Clark NW Kinter MT Elliott MH Freeman WM. Functional changes in the neural retina occur in the absence of mitochondrial dysfunction in a rodent model of diabetic retinopathy. J Neurochem (2017) 143:595–608. doi: 10.1111/jnc.14216 28902411 PMC5693713

[7] Avidor D Loewenstein A Waisbourd M Nutman A. Cost-effectiveness of diabetic retinopathy screening programs using telemedicine: a systematic review. Cost Eff Resour Alloc (2020) 18:16. doi: 10.1186/s12962-020-00211-1 32280309 PMC7137317

[8] Thapa R Bajimaya S Sharma S Rai BB Paudyal G. Systemic association of newly diagnosed proliferative diabetic retinopathy among type 2 diabetes patients presented at a tertiary eye hospital of Nepal. Nepal J Ophthalmol (2015) 7:26–32. doi: 10.3126/nepjoph.v7i1.13163 26695602

[9] Samadi Aidenloo N Mehdizadeh A Valizadeh N Abbaszadeh M Qarequran S Khalkhali H. Optimal glycemic and hemoglobin A1c thresholds for diagnosing diabetes based on prevalence of retinopathy in an Iranian population. Iran Red Crescent Med J (2016) 18:e31254. doi: 10.5812/ircmj.31254 27781118 PMC5065709

[10] Pranata R Vania R Victor AA. Statin reduces the incidence of diabetic retinopathy and its need for intervention: A systematic review and meta-analysis. Eur J Ophthalmol (2021) 31:1216–24. doi: 10.1177/1120672120922444 32530705

[11] Fundus pathology group of ophthalmology Society of Chinese Medical Association. Clinical guidelines for the diagnosis and treatment of diabetic retinopathy in China. Chin J Ophthalmol (2014) 50:851–65.

[12] Lin S Ramulu P Lamoureux EL Sabanayagam C. Addressing risk factors, screening, and preventative treatment for diabetic retinopathy in developing countries: a review. Clin Exp Ophthalmol (2016) 44:300–20. doi: 10.1111/ceo.12745 26991970

[13] Four risk factors for severe visual loss in diabetic retinopathy. The third report from the Diabetic Retinopathy Study. The diabetic retinopathy study research group. Arch Ophthalmol (1979) 97:654–5. doi: 10.1001/archopht.1979.01020010310003 426679

[14] Chung YR Lee SY Kim YH Byeon HE Kim JH Lee K. Hyperreflective foci in diabetic macular edema with serous retinal detachment: association with dyslipidemia. Acta Diabetol (2020) 57:861–6. doi: 10.1007/s00592-020-01495-8 32114640

[15] Do JR Park SJ Shin JP Park DH. Assessment of hyperreflective foci after bevacizumab or dexamethasone treatment according to duration of macular edema in patients with branch retinal vein occlusion. Retina (2021) 41:355–65. doi: 10.1097/IAE.0000000000002826 32349101

[16] Yu Y Zhang J Zhu R Zhao R Chen J Jin J. The profile of angiogenic factors in vitreous humor of the patients with proliferative diabetic retinopathy. Curr Mol Med (2017) 17:280–6. doi: 10.2174/1566524017666171106111440 29110608

[17] Schreur V Altay L Van Asten F Groenewoud JMM Fauser S Klevering BJ. Hyperreflective foci on optical coherence tomography associate with treatment outcome for anti-VEGF in patients with diabetic macular edema. PloS One (2018) 13:e0206482. doi: 10.1371/journal.pone.0206482 30379920 PMC6209345

[18] Do DV Nguyen QD Vitti R Berliner AJ Gibson A Saroj N. Intravitreal aflibercept injection in diabetic macular edema patients with and without prior anti-vascular endothelial growth factor treatment: outcomes from the Phase 3 program. Ophthalmology (2016) 123:850–7. doi: 10.1016/j.ophtha.2015.11.008 26832658

[19] Zhen C Amin X Qinyun X. Efficacy of intravitreal abiercept in the treatment of diabetic macular edema. J Wuhan Univ (Med Ed) (2019) 40:842–4.

[20] Qianyi L. Clinical efficacy of abiercept via intravitreal injection in diabetic macular edema. New Adv Ophthalmol (2019) 39:340–2.

[21] Avitabile T Azzolini C Bandello F Boscia F De Falco S Fornasari D. Aflibercept in the treatment of diabetic macular edema: a review and consensus paper. Eur J Ophthalmol (2017) 27:627–39. doi: 10.5301/ejo.5001053 29077188

[22] Lu L Jun LJ Changzheng C. Long-term follow-up of continuous intravitreal injection of diabetic macular edema. J Wuhan Univ (Med Ed) (2020) 41:819–23.

[23] Rebecca SFF Shaikh FF Jatoi SM. Comparison of efficacy of combination therapy of an intravitreal injection of bevacizumab and photocoagulation versus Pan Retinal Photocoagulation alone in High risk Proliferative Diabetic Retinopathy. Pak J Med Sci (2021) 37:157–61. doi: 10.12669/pjms.37.1.3141 PMC7794115 33437269

[24] Zhao T Chen Y Liu D Stewart JM. Optical coherence tomography angiography assessment of macular choriocapillaris and choroid following panretinal photocoagulation in a diverse population with advanced diabetic retinopathy. Asia Pac J Ophthalmol (Phila) (2020) 10:203–7. doi: 10.1097/APO.0000000000000345 33181550

[25] Lorusso M Milano V Nikolopoulou E Ferrari LM Cicinelli MV Querques G. Panretinal photocoagulation does not change macular perfusion in eyes with proliferative diabetic retinopathy. Ophthalmic Surg Lasers Imaging Retina (2019) 50:174–8. doi: 10.3928/23258160-20190301-07 30893451

[26] Li X Xie J Zhang L Cui Y Zhang G Wang J. Differential distribution of manifest lesions in diabetic retinopathy by fundus fluorescein angiography and fundus photography. BMC Ophthalmol (2020) 20:471. doi: 10.1186/s12886-020-01740-2 33261573 PMC7709243

[27] Komiyama M Takanabe R Ono K Shimada S Wada H Yamakage H. Association between monocyte chemoattractant protein-1 and blood pressure in smokers. J Int Med Res (2018) 46:965–74. doi: 10.1177/0300060517723415 PMC5972233 29098933

[28] Dong L Lv XY Wang BJ Wang YQ Mu H Feng ZL. Association of monocyte chemoattractant protein-1 (MCP-1)2518-A/G polymorphism with proliferative diabetic retinopathy in northern Chinese type 2 diabetes. Graefes Arch Clin Exp Ophthalmol (2014) 252:1921–6. doi: 10.1007/s00417-014-2651-1 24809310

[29] Ambros V. MicroRNA pathways in flies and worms: growth, death, fat, stress, and timing. Cell (2003) 113:673–6. doi: 10.1016/s0092-8674(03)00428-8 12809598

[30] Liu R Liu CM Cui LL Zhou L Li N Wei XD. Expression and significance of MiR-126 and VEGF in proliferative diabetic retinopathy. Eur Rev Med Pharmacol Sci (2019) 23:6387–93. doi: 10.26355/eurrev_201908_18518 31378876

